# Research on Aseptic Milk Extraction Technology and Mechanism of Slightly Acidic Electrolytic Water Coupled with Ultrasound Treatment

**DOI:** 10.3390/foods14101711

**Published:** 2025-05-12

**Authors:** Ye Liu, Qinggang Xie, Dongying Cui, Jiaqi Ren, Wanyu Zhao, Xiaoxi Xu

**Affiliations:** 1 College of Food Science, Northeast Agricultural University, Harbin 150030, China; l2283993870@163.com (Y.L.);; 2 Heilongjiang Firmus Dairy Co., Ltd., C-16,10A Jiuxianqiao Rd., Chaoyang, Beijing 100015, China

**Keywords:** slightly acidic electrolyzed water, ultrasound, microorganisms, milking system

## Abstract

The use of low-temperature antibacterial technology is a processing method designed to preserve the biological activity of milk to the greatest extent. Traditional feeding and milking practices result in high levels of microbiological contamination of raw milk after extraction, mainly from cows and milking equipment, especially rubber cups. Ultrasonic treatment combined with antimicrobial agents combine cleaning and antibacterial technology, compared with traditional cleaning methods, more efficiently and in a environmentally friendly way. In this study, the technique was demonstrated to significantly reduce the total amount of bacteria in raw milk through simulation experiments on the surface of milking cups. It was shown that ultrasound-coupled slightly electrolytic water has a good potential for application in reducing bacterial contamination in the milk extraction process on farms. We investigated the synergistic mechanism of ultrasound (US) and slightly acidic electrolytic water (SAEW) and verified the bactericidal effect of milking cups. A 20 s treatment of milking cups with US (100 W) and SAEW (90 mg/L) led to an antibacterial rate of over 90%. The bactericidal mechanism causes fragmentation of the cell membrane of pathogenic bacteria, exudation of their intracellular contents such as nucleic acids and proteins, and increases in ROS.

## 1. Introduction

As the dairy industry expands, both producers and consumers are demanding higher-quality milk. To obtain milk products with superior quality and activity, low-temperature antibacterial technology has emerged as a key area of research and development in dairy processing. Microorganisms in raw cow’s milk produced through traditional feeding and milking methods primarily originate from the environment, the cow’s body, and the milking equipment. Notably, in mechanical milking methods, the rubber cup serves as a significant source of contamination following raw milk extraction. The milking cups are in direct contact with the cow’s udder and teats, and if the equipment is not properly cleaned, the residual emulsion can serve as a breeding ground for microorganisms. Typical microorganisms of milking equipment present bacteria such as *Escherichia coli*, *Staphylococcus aureus*, *Listeria monocytogenes*, *Salmonella* spp., *Micrococcus* spp., *Campylobacter jejuni*, *Enterococcus faecalis*, *Citrobacter freundii* [1]. During the milking process, the rubber inflatable liner inside the stainless steel housing of the milking cup pulsates under the influence of an electric current, causing the milk to flow [2]. The rubber cup has a limited service life [3], and when it becomes overdue for use, cracks resulting from rubber aging can create a reservoir for milk, promoting bacterial growth and reproduction. This contamination leads to a significant increase in the total bacterial count, particularly during the spring and summer seasons [4].

Currently, food processing antibacterial methods are primarily categorized into chemical, physical, and biological methods [5]. Factories often use chemicals, such as iodophor solution, for sterilizing milking cups, which may increase the level of chemical residues in the milk [6,7]. In the food industry, traditional heat treatments such as autoclaving and pasteurization may cause unavoidable adverse effects, including the loss and inactivation of heat-sensitive nutrients and biologically active substances in food, such as vitamins (e.g., B-vitamins), functional proteins (e.g., lactoferrin), and certain enzyme activities [8,9,10]. These changes can, in turn, affect the nutritional value and functionality of the food [11]. In addition, during the high temperature antibacterial process, certain chemical reactions can lead to changes in the composition of the food, such as fat oxidation at high temperatures and the Maillard reaction, which can produce off-flavors and harmful substances. To ensure food safety, reducing the number of microorganisms in raw milk is a fundamental prerequisite for the application of low-temperature antibacterial and preservation technologies. Therefore, it is essential to effectively control and minimize the biological contamination of raw milk during the milking process. Cleaning the milking cups reduces the bacterial count in the raw milk, helping to reduce the risk of contamination from one cow to another. In order to solve these problems, safer and quicker methods of cleaning milk cups for pasture-pressed milk should be sought.

Acid electrolytic water (AEW) is considered an environmentally friendly disinfectant and has been widely used for disinfection in the healthcare, food processing, and environmental sanitation industries [12,13] due to its low pollution and minimal impact on human health [14,15]. Slightly acidic electrolytic water (SAEW), with a pH range of 5.0 to 6.5, reduces the surface corrosion of food processing equipment and minimizes potential threats to human health and the environment [16,17]. Currently, SAEW is effectively used as an antibacterial agent in vegetables, fruits, and aquatic products, providing a safer method for eliminating bacteria [18,19]. For on-farm applications, SAEW at 60 mg/L was effective in removing bacteria from on-farm milking cups [20], but the processing time is long and needs to be more efficient before it can be put into practical use. After SAEW treatment, the protective barriers (cell wall and cell membrane) of bacteria are disrupted, leading to an increase in cell membrane permeability [21]. After SAEW diffuses through the membrane, hypochlorous acid and the generated reactive oxygen species (ROS) induce a complex series of changes in intracellular metabolites [22]. SAEW is ineffective in inhibiting Gram-negative foodborne pathogens [23,24], highlighting the urgent need to explore alternative methods to synergize the action of SAEW and enhance antimicrobial efficacy. In contrast, ultrasonic inactivation of microorganisms has been applied to fresh fruits and vegetables, frozen foods, and dairy products [25], which typically retain most of their natural nutrients and flavor. Ultrasonic treatment is milder than traditional thermal inactivation methods, allowing food products to retain their organoleptic and nutritional properties to the greatest extent possible [26,27]. Ultrasonication disrupts the cell wall primarily through cavitation, with the increase in local temperature and pressure, along with the production of free radicals, contributing to the inactivation of microorganisms [28,29,30]. Therefore, the combined antibacterial technique of ultrasound and SAEW, which enhances the efficacy of disrupting cellular structures, is expected to become a more promising and efficient method in the future. A study confirmed that the synergistic effect of US (500 W) and SAEW (50 mg/L) effectively reduced the decay rate of sweet potato cubes compared to SAEW treatment alone [31]. Similarly, Luo [32] observed that the SAEW + US + 40 °C synergistic treatment significantly reduced Bacillus cereus on potatoes within 3 min. Rapid antibacterial processes using low-power ultrasound combined with acidic electrolytic water have not been reported, and existing studies on ultrasound combined with acidic electrolytic water primarily focus on fresh fruits, vegetables, and refrigerated foods [23,33,34,35].

This study aims to take two Gram-negative and two Gram-positive pathogenic bacteria commonly found in milk as target strains, and explore the bactericidal effect by using micro-acid electrolyzed water coupled with ultrasonic technology, revealing the bactericidal mechanism. It will also verify and analyze the bactericidal effect on the residual bacteria on the surface of the milking cups used in the dairy farm. This research can provide a scientific basis for the antibacterial mechanism of micro-acid electrolyzed water coupled with ultrasonic technology, and lay a theoretical and application foundation for the development of future milking cleaning technologies and equipment as well as low-temperature sterilized products.

## 2. Materials and Methods

### 2.1. Bacterial Strains and Reagents

*Escherichia coli* (*E. coli*), *Salmonella Enteritidis* (*S. enteritidis*), *Staphylococcus aureus* (*S. aureus*), and *Listeria monocytogenes* (*L. monocytogenes*) used in this experiment were obtained from the Heilongjiang Provincial Centre for Disease Control and Prevention. *E. coli*, *S. enteritidis* and *S. aureus* were cultured in lysogeny broth (LB) medium, and *Listeria monocytogenes* was cultured in Brain Heart Infusion (BHI) medium at 37 °C for 12 h. Cells were collected, centrifuged at 6000× *g* for 10 min, and resuspended in 0.9% NaCl.

### 2.2. SAEW and US Preparation

SAEW was prepared using an acidic water electrolyzer (Beijing Intercontinental Resources and Environmental Protection Technology Co., Beijing, China). Preparation requires 2 L of a 1 g/L NaCl solution, to which 0.35 mL of concentrated HCl was added, and the mixture was electrolyzed for 2.5 h to obtain SAEW with a pH of 5.3 and an available chlorine concentration (ACC) of 200 mg/L. The preparation of SAEW was carried out in the same manner as that of NaCl. The desired ACC was obtained by dilution with sterilized deionized water (DW). For sonication, a probe-based sonicator (Scientz-II D; Ningbo Scientz, Ningbo, Zhejiang, China) was used.

### 2.3. Application Effect on Milk-Pressing Cups

For the determination of the germ removal effect of a single pathogen, food-grade milking cups were submerged in glass containers filled with milk, so that the rubber inner wall of the cups came into full contact with the milk to simulate a milking scenario. We inoculate 100 μL of each of the four bacterial suspensions in a glass container with milk, and incubate at 37 °C for 3 h to allow the bacteria to colonize the inner wall of the rubber. The milking cups were removed and washed three times with sterilized deionized water (DW) and the cleaned milking cups were treated with US, SAEW, SAEW + US, and milking cups immersed in milk and not inoculated with bacteria were used as a control group. A sterile cotton swab was used to wipe off the inner wall of the squeezing bra cup and immersed in a test tube containing PBS solution vortexed and diluted to the appropriate concentration according to the gradient dilution method, and 100 μL of the liquid was taken for plate coating and incubated at 37 °C for 24 h for colony counting.

### 2.4. Determination of Minimum Inhibitory Concentration (MIC)

*E. coli*, *S. enteritidis*, *S. aureus*, and *L. monocytogenes* cultured to the logarithmic phase of growth were inoculated at 1% in sterile LB and BHI liquid medium. After mixing, SAEW was added so that the ACC in the solution was 200 mg/L. The bacterial solution was sequentially diluted so that the ACC of SAEW within the solution was 50, 100, and 150 mg/L, respectively. The samples without SAEW were used as a blank control group; 200 µL of each sample was mixed and added into 96-well plate to determine the change in OD 600 absorbance value over time, the measurement time was 48 h at 37 °C, and the growth cycle was measured every 1 h to determine the growth cycle. The lowest SAEW concentration for aseptic growth was used as the MIC.

### 2.5. Study on the Effect of Combined Sterilization

The experiment was conducted in four groups: control (no treatment), SAEW-treated, US-treated, and SAEW + US-treated. For the SAEW treatment group, 9 mL of SAEW was added to a sterile glass test tube, followed by 1 mL of bacterial culture. For the US treatment group, in this experiment, ultrasonic waves with a power of 100 w were used for ultrasonic treatment using an ultrasonic probe with a diameter of 6 mm to treat the bacterial suspensions of the four groups for 10, 20, and 30 s. The ultrasonic waves were used to treat the bacterial suspensions of the four groups. When ultrasound alone is used to treat bacteria, it has little or no bactericidal effect, and some studies have shown that the bactericidal effect is not obvious when ultrasound alone is used to treat bacteria [36,37]. This is mainly attributed to the fact that the mechanical effect produced by ultrasound is weak and not sufficient to cause bacterial inactivation. We filled a sterile glass test tube with 9 mL of sterile deionized water (DW) and added 1 mL of bacterial culture, with the probe immersed at least 2 cm below the surface of the liquid. For the SAEW + US treatment group, a volume of 9 mL of SAEW was transferred to a sterile test tube and 1 mL of bacterial culture (about 8.0 log CFU/mL) was immediately added to the test tube, mixed well, and then sonicated, with the US fixed at 100 W. The test tubes were then subjected to the same treatment procedure as the SAEW + US treatment group. After treatment, 1 mL of each sample was transferred to a test tube containing 9 mL of neutralization buffer (0.5% Na_2_S_2_O_3_) and shaken well. After neutralization for 5 min, serial dilutions were made with sterile 0.1% peptone water and the dilutions (0.1 mL) were inoculated on agar medium and then incubated at 37 °C for 24 h to identify the surviving bacteria. The bactericidal effects of the different treatments were compared in two steps. Firstly, four groups of bacterial suspensions were treated with three different ACC (80, 90, and 100 mg/L) at fixed time (30 s). After comparing the selected bactericidal methods, the ACC (90 mg/L) was fixed and the four groups of bacterial suspensions were treated at different times (10, 20, and 30 s).

### 2.6. Scanning Electron Microscope (SEM) Analysis

Each sample was fixed with 2.5% glutaraldehyde at 4 °C for 12 h and allowed to dry; the upper spacers were removed and the slides were cut using a glass cutter according to the grid in which the samples were placed and then fixed for 90 min with 1% (*v*/*v*) osmic acid, followed by dehydration with a gradient of ethanol at concentrations of 30, 50, 80, 90, and 100% each time for 10 min, and then washed with 100% tert-butanol. The treated slides were sprayed with gold for SEM (S-3400 N, Hitachi, Ltd., Tokyo, Japan) observation.

### 2.7. Determination of Nucleic Acid and Soluble Protein Release

The treated bacterial suspension was centrifuged at 6000× *g* for 10 min at 4 °C, and the bacterial supernatant was collected. Nucleic acids and soluble proteins released from the four bacteria were measured by determining the absorbance of the supernatant at 260 nm and 280 nm using a UV–visible spectrophotometer (Beijing Purkinje Co., Beijing, China).

### 2.8. Determination of Reactive Oxygen Species (ROS)

The ROS assay kit (Beyotime Bioengineering Institute, Shanghai, China) was used according to the manufacturer’s instructions to measure the intracellular ROS levels. The samples were centrifuged at 2000× *g* and allowed to stand for 5 min. The samples were resuspended in 10 μM DCFH-DA working solution and incubated in the dark at 37 °C for 30 min. We inverted the mixing every 3–5 min to ensure full contact between the probe and the cells. Fluorescence intensity was measured using 495 nm excitation wavelength and 530 nm emission wavelength, using a Multi-mode Microplate Reader (Thermo Fisher Scientific (China) Co., Ltd., Shanghai, China).

### 2.9. Flow Cytometry Test

Using the treated bacterial fluids, the organisms were stained with PI (Beyotime Bioengineering Institute, Shanghai, China), collected by centrifugation at 10,000× *g* for 1 min to remove excess fluorescent probe, and resuspended in PBS buffer. For the flow cytometry assay, the fluorescence in the cells was detected separately and then converted to a digital signal, and the sample fluorescence was detected at 620 nm.

### 2.10. Examination of Cell Membrane Permeability

Cell membrane permeability was determined by measuring the activity of β-galactosidase in bacteria entering the culture medium using ONPG (Beyotime Bioengineering Institute, Shanghai, China) as a substrate for the reaction. After centrifugation of the treated bacterial suspension, the supernatant was taken and ONPG (final concentration of 1.5 mmol/L) was added and incubated at 37 °C for 30 min, and the absorbance value of its product, o-nitrophenol, at 420 nm was determined [38].

### 2.11. Laser Scanning Confocal Microscopy (LSCM) Analysis

The cell membrane permeability assay used the fluorescent probes cFDA and PI (Beyotime Bioengineering Institute, Shanghai, China) to distinguish between live and dead cells. The logarithmic phase of the bacterial solution was centrifuged at 5000× *g* for 10 min, and the supernatant was removed and washed once with 0.85% NaCl. The bacterial solution was treated by different methods, and the sample with the same volume of 0.85% NaCl added was used as a blank control group, which was kept at room temperature for 1 h and shaken every 15 min. Bacteria were collected by centrifugation and suspended in 500 μL of 0.85% NaCl, and the fluorescent probe cFDA was added to each sample to reach a final concentration of 100 μmol/L, and kept away from light for 10 min; then, 10 μL of red nucleic acid fluorescent staining solution PI was added to each sample to reach a final concentration of 30 μmol/L, and the reaction was kept away from light for 10 min. The mixture was centrifuged and the slime was suspended in 500 μL of saline, 3 μL of the slime was placed on a slide with a coverslip, and it was observed under a laser confocal microscope and photographed.

### 2.12. Statistical Analysis

All data were analyzed with Origin 2021 software (Originalab, Northampton, MA, USA). Analysis of variance (ANOVA) was used to analyze various factors (treatment, ACC, and time). In addition, Tukey’s test assessed differences between means, with *p* < 0.05 representing significant differences. In characterizations and comparisons of bacterial counts involving multiple intestinal samples, the logarithmic transformation should be used.

## 3. Results

### 3.1. Milking Cups Simulate the Antibacterial Effect

**Figure 1:**
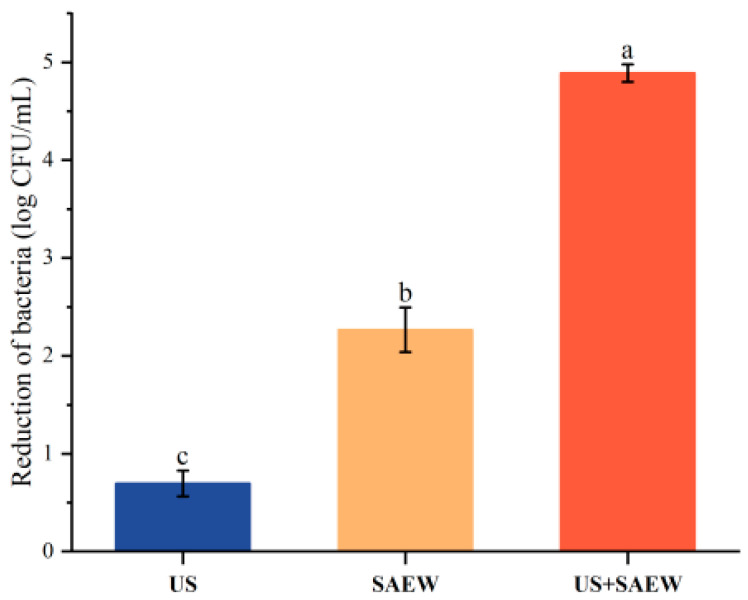
Sterilization of the surfaces of milk-pressing cups after immersion in milk mixed with *E. coli*, *S. enteritidis*, *S. aureus*, and *L. monocytogenes* and after treatment with US, SAEW, US + SAEW. Values are the mean of triplicate measurements ± standard error; different letters indicate significant differences (*p* < 0.05).

The initial bacterial count was 5.01 ± 0.15 CFU/mL after the four pathogenic bacteria were mixed and treated. The reduction in bacterial count during treatment with US, SAEW, and SAEW + US is shown in Figure 1, with reductions of 0.69 ± 0.13, 2.67 ± 0.23, and 4.89 ± 0.09 CFU/mL, respectively. The results showed that the US treatment group had a lesser reduction in microorganisms on the surface of milk-pressing cups, while the SAEW treatment group had a significant effect on the killing of pathogenic bacteria (*p* < 0.05). The bactericidal effect was more significant when the two were combined and US and SAEW acted synergistically (*p* < 0.05). After a preliminary simulation of the antibacterial surface of the cups, it can be seen that the SAEW + US antibacterial effect is good and can kill more than 95% of the microorganisms on the rubber surface in a short period of time, which proves that the method has research significance.

### 3.2. Minimum Inhibitory Concentration (MIC) Measurements for SAEW

**Figure 2:**
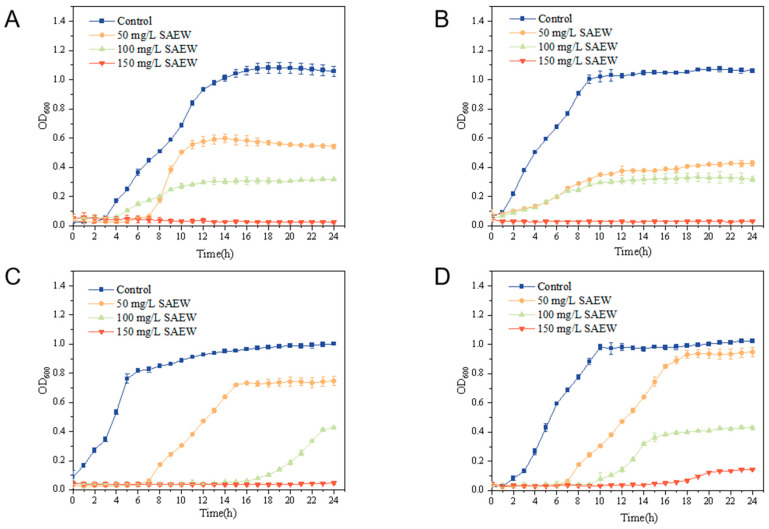
Growth curves of *E. coli* (**A**), *S. enteritidis* (**B**), *S. aureus* (**C**), and *L. monocytogenes* (**D**) in different concentrations of slightly acidic electrolytic water.

At different concentrations of the bacterial solution, absorbance at 600 nm varies, reflecting the bacterial growth state. The absorbance of the bacterial solution is measured every hour, and the average value is used to construct the growth kinetic curve. As shown in Figure 2, SAEW, at a concentration of 50 mg/L, inhibited all four pathogenic bacteria during the first 6 h. After 6 h, the bacteria had proliferated significantly; 100 mg/L SAEW had an inhibitory effect on the bacteria, but could not inhibit the growth of the bacteria completely, though 150 mg/L SAEW could inhibit the bacteria completely; therefore, the MIC of SAEW was 150 mg/L for this. The inhibitory effect of each concentration of SAEW on *L. monocytogenes* was analyzed by its growth curve, and it was found that 150 mg/L SAEW remained completely inhibitory to the bacteria for 20 h when measuring the absorbance value at OD 600 nm; 100 mg/L SAEW had a smaller inhibitory effect on *L. monocytogenes*, and the concentration of 50 mg/L SAEW had almost no inhibitory effect at all. Therefore, the MIC of SAEW against *L. monocytogenes* was higher than the other three bacteria.

### 3.3. Bactericidal Efficacy of SAEW, US, and SAEW + US on the Tested Strains

**Figure 3:**
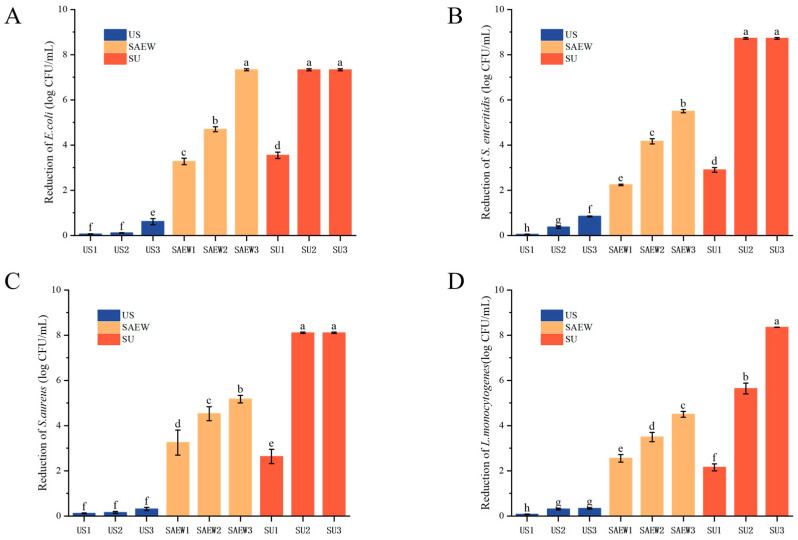
Inactivation of *E. coli* (**A**), *S. enteritidis* (**B**), *S. aureus* (**C**) and *L. monocytogenes* (**D**) by different treatments: US, ultrasound (100 W) treatment at different times (10, 20, and 30 s); SAEW, SAEW treatment at different ACCs (80, 90, and 100 mg/L) for 20 s; and SAEW + US, ACC (90 mg/L) fixation for four groups of bacterial suspensions at different times (10, 20, and 30 s) for the four groups of bacterial suspensions. Values are the mean of triplicate measurements ± standard error; different letters indicate significant differences (*p* < 0.05).

In this study, a one-way experimental design was used to compare the inhibitory effects of SAEW, US, and SAEW + US on the four tested strains. Initial counts of *S. enteritidis*, *S. aureus*, *E. coli*, and *L. monocytogenes* were 8.72 ± 0.04, 8.11 ± 0.03, 7.33 ± 0.05, and 8.35 ± 0.01 log CFU/mL, respectively. It has been shown that the bactericidal effect of US and SAEW is related to the bacterial species; e.g., SAEW alone is ineffective in treating S. aureus [39]. Inactivation of all four strains by US alone after different times (10, 20, and 30 s) was not significant. As shown in Figure 3A, the amount of *E. coli* in the suspension was reduced by approximately 4.69 log CFU/mL after treatment with 90 mg/L of SAEW for 30 s. The antimicrobial effect of SAEW on the other three strains was similar; i.e., the inactivation effect of SAEW (80, 90, and 100 mg/L) was significantly better than that of the US treatment for the same time (30 s) (*p* < 0.05). The antimicrobial effect of each treatment group increased with increasing ACC and time, where the number of bacteria treated with 100 mg/L of SAEW was significantly reduced and almost 90% of the bacteria died. At the same concentration of SAEW and treatment time, the inhibitory effect of different treatments was SAEW + US > SAEW > US. The results showed that *S. enteritidis*, *S. aureus*, and *E. coli* were inactivated and *L. monocytogenes* was reduced by more than 90% after 20 s of treatment at an ACC of 90 mg/L and a US of 100 W. The results of the study showed that the combination of US and SAEW had a good bactericidal effect against both Gram-positive and Gram-negative bacteria.

### 3.4. Morphological Changes in Cells

**Figure 4:**
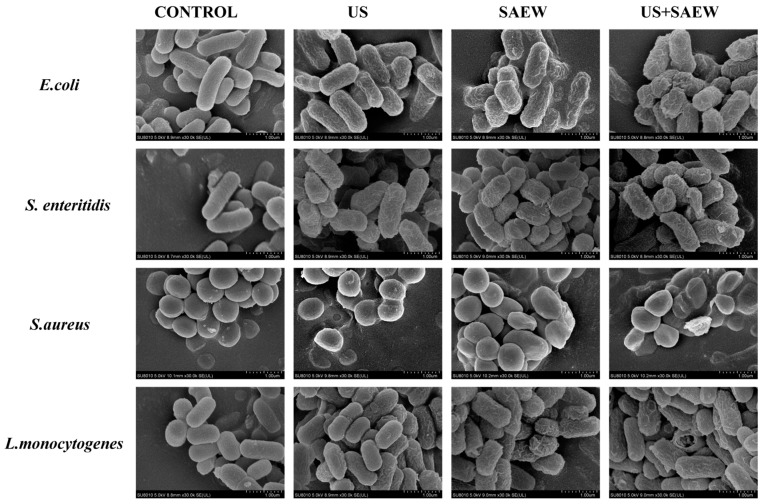
Scanning electron microscopy images of *E. coli*, *S. enteritidis*, *S. aureus*, and *L. monocytogenes* after US, SAEW, and US + SAEW treatments.

The results, as shown in Figure 4, scanning electron microscopy (SEM) was used to observe the morphological changes in the four bacteria under different treatments (SAEW, US and SAEW + US). *E. coli*, *S. enteritidis*, and *L. monocytogenes* without any treatment had smooth surfaces with intact cells and normal rod-like structures. Untreated *S. aureus* had a smooth surface with intact, regular spherical cells. After US treatment, the surfaces of *E. coli* and *S. enteritidis* were slightly wrinkled, and there were no significant changes in *S. aureus* and *L. monocytogenes*, with only a few cells collapsing in morphology. This may be due to the fact that Gram-positive bacteria have thicker cell walls than Gram-negative bacteria, which are less likely to change cell morphology in the face of ultrasound treatment. The morphology of the SAEW-treated *E. coli* was collapsed and deformed, and the other three bacteria showed similar results. The SAEW + US treatment was significantly more effective, resulting in the most severe cellular damage, with significant rupture of cell membranes and leakage of large quantities of intracellular compounds in all four strains of the test organisms.

### 3.5. Determination of Nucleic Acid and Soluble Protein Release

**Figure 5:**
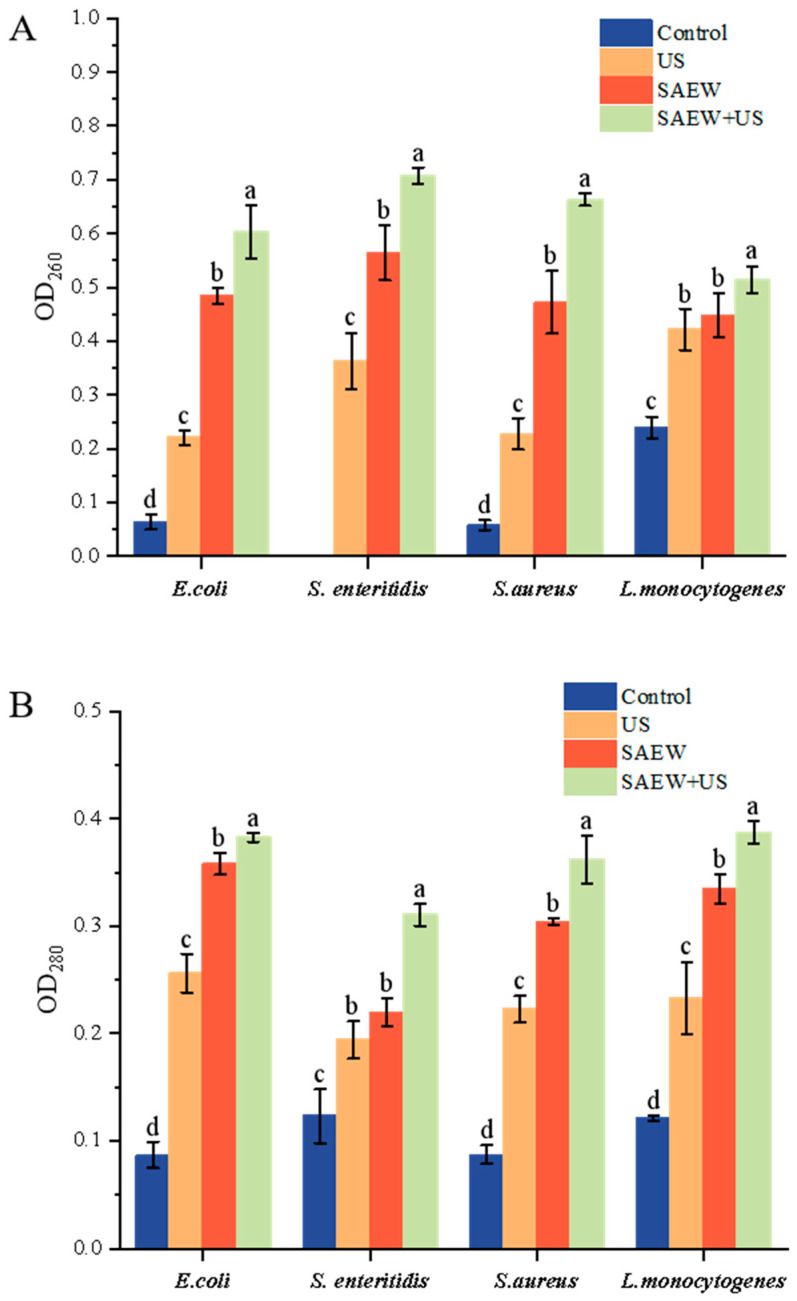
Determination of nucleic acid (**A**) and soluble protein (**B**) release from *E. coli*, *S. enteritidis*, *S. aureus*, and *L. monocytogenes* after treatment with US, SAEW, and US + SAEW. Values are the mean of triplicate measurements ± standard error; different letters indicate significant differences (*p* < 0.05).

Further measurements of cell membrane damage are required to verify that the bacteria ruptured completely in the combined treatment. OD 260 nm and OD 280 nm were used to determine the effect of different treatments on the leakage of nucleic acids and proteins from the cells of the four bacterial species; excessive leakage of nucleic acids and proteins can lead to bacterial death, and therefore this indicator is considered to be related to cellular damage [14]. The results, as shown in Figure 5, showed that the release of nucleic acids and proteins was significantly higher in SAEW-treated cells compared with the control group (*p* < 0.05). All treatments resulted in the release of intracellular UV-absorbed material, with greater leakage of intracellular material in the SAEW treatment than in the US treatment. Nucleic acid and protein leakage was higher in the four bacteria under SAEW + US treatment than US and SAEW alone. Both the damaged cell membranes and the pores seen under the electron microscope may have contributed to the increased leakage of cell contents, which is consistent with the scanning electron microscopy observations.

### 3.6. Changes in Cellular ROS Levels

**Figure 6:**
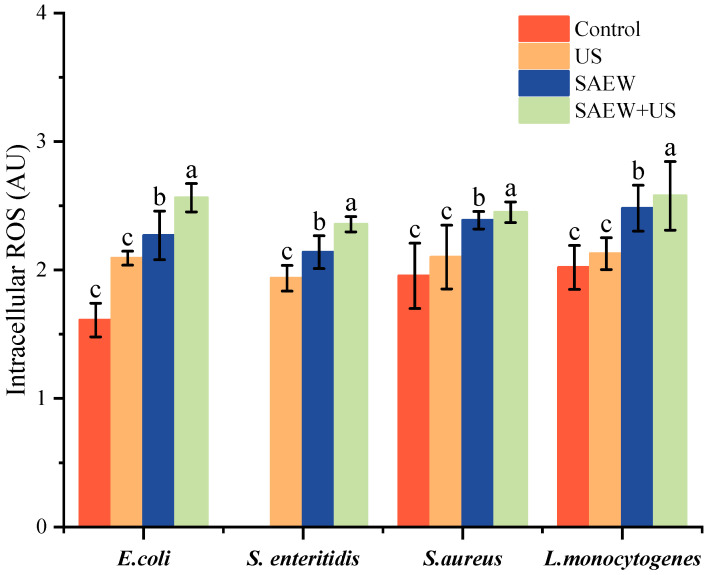
Effect of different treatments on the production of reactive oxygen species by pathogenic bacteria. Values are the mean of triplicate measurements ± standard error; different letters indicate significant differences (*p* < 0.05).

Changes in ROS levels are considered to be another indicator of cell membrane damage correlation. Excessive production of ROS by cells causes cellular oxidative stress, leading to cell membrane damage and cell death [40]. As shown in Figure 6, no significant difference in reactive oxygen species (ROS) accumulation was observed between the single US treatments relative to the control (*p* < 0.05). ROS accumulated in both the SAEW treatment group and the SAEW + US treatment (*p* < 0.05). SAEW may be a determinant of increased ROS accumulation, it has been shown that HClO in SAEW enters the cells to cause oxidative stress, and SAEW treatment causes ROS accumulation in both *Listeria monocytogenes* and *Salmonella enterica* [41,42], which is consistent with the experimental results of this study. In addition to HClO, another factor in ROS accumulation is -OH, which reacts with cell membranes in a lipid peroxidation reaction. The SAEW + US-treated group had the highest ROS accumulation, which was significantly higher than the SAEW-treated group, suggesting that the synergistic treatment of SAEW + US, at the same time, triggered a greater burst of ROS in the four bacterial species, leading to extensive cell membrane damage and cell death.

### 3.7. Analysis of Cell Membrane Integrity

**Figure 7:**
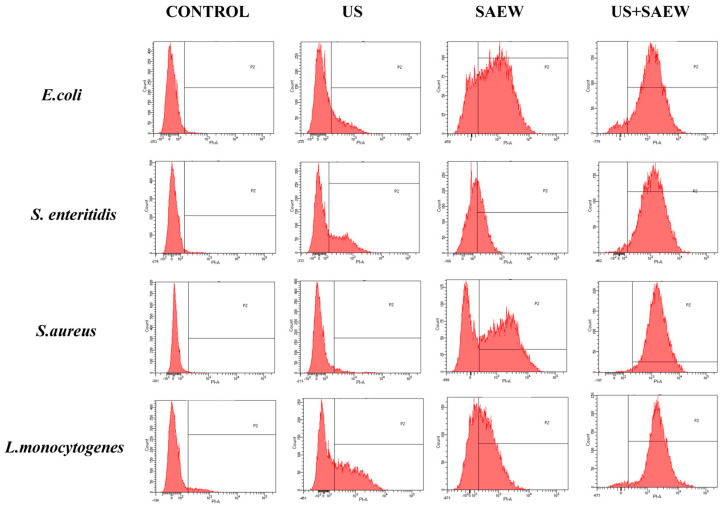
The effect of different treatments on cell death was detected by flow cytometry.

Flow cytometry was used to determine bacterial mortality by detecting cell membrane integrity in the four experimental strains using the PI method, in which a staining reagent, PI, crosses damaged cell membranes and binds to nucleic acids for staining [43]. The x-axis in the graph indicates the level of penetration, and the more propidium iodide binds to the DNA, the more the peak is shifted to the right of the x-axis. As shown in Figure 7, the experimental results showed that the percentage of positive bacteria in the control group was much lower than that of the other three treated groups, the surface cell membrane remained intact, and PI could not cross the intracellular membrane to bind to DNA. The proportions of positive areas for *E. coli*, *S. enteritidis*, *S. aureus*, and *L. monocytogenes* in the co-treatment were 98.1%, 95.1%, 96.2%, and 90.1%, respectively, suggesting that more than 90% of the cells of the four causative organisms were destroyed, which is in agreement with the results of bacterial inactivation. The number of deaths of *S. aureus* and *L. monocytogenes* after ultrasound treatment was less than those of *E. coli* and *S. enteritidis*, and this difference may be due to different Gram staining. Gram-positive bacteria are thicker than Gram-negative bacteria and ultrasound is less effective, as confirmed in the study by Katherine [25] et al. SAEW treatment alone resulted in the death of most cells and was effective for all four experimental strains, whereas, under combined treatment, both Gram-positive and Gram-negative bacteria showed a significant increase in the intensity of their fluorescence, and shifted towards longer wavelengths. The above results showed that the combined treatment of US and SAEW caused irreversible cell destruction, further demonstrating that the combined treatment of US and SAEW has a bactericidal effect.

### 3.8. Analysis of Cell Membrane Permeability

β-galactosidase is an intracellular glycoside hydrolase that hydrolyses lactose to galactose and glucose, with lactose as an inducer, and is often used as a biomarker of intracellular membrane permeability. Extracellular β-galactosidase is extremely low when the cell membrane is structurally intact, and it is only when the intracellular membrane is damaged that ONPG can intervene inside the cell and react with intracellular β-galactosidase to produce yellow o-nitrophenol. There was no significant difference in extracellular β-galactosidase secretion activity between the control and US-treated groups, suggesting that the extent of US damage to cell membranes was low. Significant differences were found in both SAEW-treated and control groups (*p* < 0.05). When SAEW and US acted synergistically, the intracellular membrane permeability tended to increase and was greater than that of the SAEW-treated group. The results showed that both SAEW and SAEW + US could affect cell membranes, but SAEW and US were more effective when they acted synergistically, which might be caused by the fact that the number of bacteria damaged by SAEW + US was more than that of SAEW alone.

**Figure 8:**
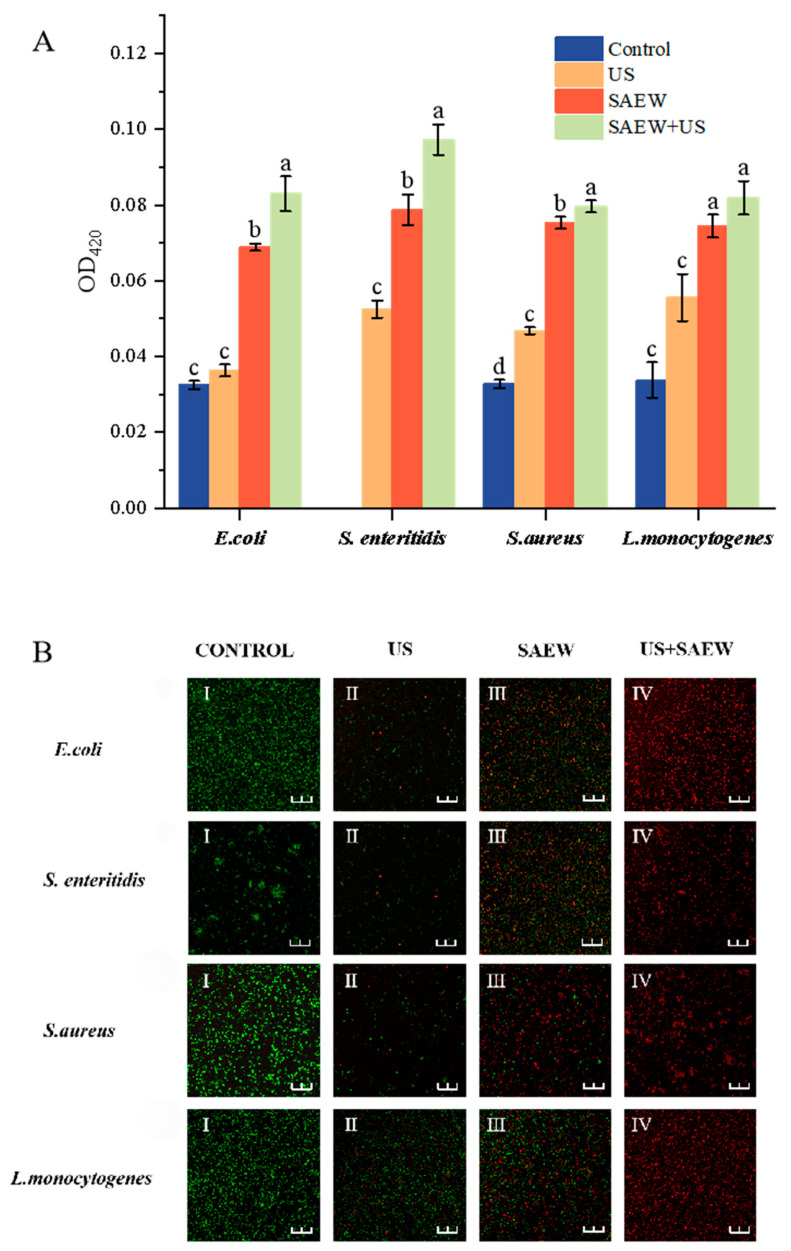
Confocal images of *E. coli*, *S. enteritidis*, *S. aureus*, and *L. monocytogenes* after US, SAEW, and US + SAEW treatment (**A**,**B**). The scale bar is 25 μm, green dots indicate bacteria with intact membranes, and red dots indicate bacteria with damaged membranes. Values are the mean of triplicate measurements ± standard error; different letters indicate significant differences (*p* < 0.05). (**I**–**IV**) represent the images of control group, US-treated group, SAEW treated group and US + SAEW treated group, respectively.

Staining by Nucleic Acid Fluorescent Probe 5(6)-cFDA and Nucleic Acid Fluorescent Probe PI showed a green color in undamaged cells and a red color in broken cells. Based on the staining results, changes in cell membrane permeability can be visualized by laser confocal microscopy. As shown in Figure 8, the proportion of red dots in cells treated with US is smaller than that in cells treated with SAEW, which indicates that the degree of cell damage by US is smaller than that by SAEW, which is consistent with the experimental results of scanning electron microscopy and flow cytometry. When US and SAEW were co-treated, the cells of all four strains were stained red, indicating that the cell membranes had been severely disrupted by the treatment. Thus, the cell membrane damage assay further confirmed the combined bactericidal mechanism of US and SAEW, and also confirmed that the inhibitory ability of SAEW was stronger than that of US.

## 4. Discussion

**Figure 9:**
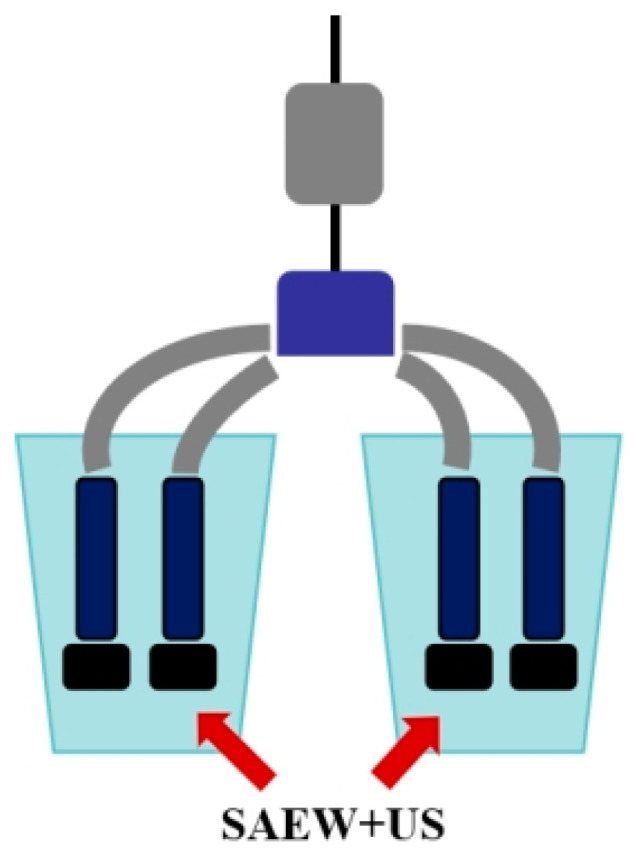
Schematic diagram of ultrasound combined with micro-acidic electrolytic water for decontamination of milking cups.

In this study, we investigated the aseptic milk extraction technique and the mechanism of bacteriostatic inhibition of this technique against four pathogenic bacteria based on the combined antibacterial technique of ultrasound and microacidic electrolytic water. Studies have shown that ultrasound combined with microacidic electrolytic water can effectively treat microorganisms adhering to the surface of squeezed breast cups (Figure 9), and the combined treatment has the advantages of more antibacterial, shorter time and higher efficiency. Antibacterial rates in excess of 90% can be achieved by treating press cups with 90 mg/L of slightly acidic electrolytic water (SAEW) in combination with 100 W of ultrasound (US) for 20 s. In the present study, SAEW combined with US treatment had a significant bactericidal effect, probably due to the rapid disruption of cell membranes in a short period of time and better penetration of SAEW into the cells to inactivate it [44]. Similar results were reported in the study by Guo [45] for combined NaOCl and US inhibition. Antibacterial ultrasonic treatment acts on the external morphology of microbial cells, mainly causing microbial surface deformation [46]. However, differences in the cell membranes of Gram-negative and Gram-positive bacteria can make the ultrasound-assisted microacidic electrolytic water bactericidal technique less effective for Gram-negative bacteria than for Gram-positive bacteria at the same parameter intensity [47]. SAEW can effectively kill bacteria due to the fact that the effective chlorine component in SAEW is able to disrupt the structure of bacterial cell membranes, leading to a large amount of intracellular material overflow, destroying intracellular nucleic acids, proteins, and other substances, and promoting cell death [48]. It is assumed that the combination of SAEW + US can reduce the effective chlorine component of SAEW to act on the cell membrane during the lethal process of microorganisms, and most effective chlorine can act directly on the proteins, nucleic acids and other substances, which can significantly enhance its bactericidal effect under the same process intensity. Under ultrasonic conditions, the bactericidal substance hypochlorous acid in SAEW changes the mass transfer property of the cell membrane and destroys the cell membrane under ultrasonic treatment, which makes it easier for ClO- and OH- to enter the cell and act directly on the intracellular substances, thus enhancing the bactericidal effect, making the synergistic bactericidal effect of SAEW + US far greater than that of single-technology bactericidal effect. The synergistic effect of ultrasound and slightly acidic electrolytic water may be due to the fact that ultrasound alters the mass-transfer properties of the cell membrane, making it easier for the active chlorine to enter the cell and react with intracellular substances, thus allowing the active chlorine to accelerate the process of disrupting the cell membrane.

The main purpose of this technological cleaning equipment process for farms is to properly clean the milking equipment after use [49], and the duration of the cycle depends on the type and amount of cleaning solution and the efficiency of the mechanical action [50]. During the cyclic cleaning process, the temperature of the cleaning solution is higher in order to dissolve the dirt attached to the milking cup in the water [51]. In this study, the SAEW cleaning effect is significant, and the US plays a role in quickly shocking the dirt adhered to the milking cup into the solution, and the synergistic effect of the two reduces the equipment-cleaning time to 20 s, which is more efficient compared to the traditional cleaning system, and provides a theoretical basis for the future addition of new cleaning systems.

## 5. Conclusions

In conclusion, the present study confirms the good synergistic effect of combined SAEW and US treatments on the inactivation of pathogenic bacteria. SAEW (90 mg/L) combined with US (100 W) can inactivate more than 90% of breastshield cups in just 20 s. Cavitation of US facilitated the entry of SAEW into the bacterial cell to disrupt the cell membrane, leading to the accumulation of ROS in the cell and the release of nucleic acids and soluble proteins, which acted as a bactericidal agent. This combined antibacterial technology proposes a new direction and provides a scientific theoretical basis for the future cleaning and sterilization of milk extraction systems in farms.

## Data Availability

The data presented in this study are available on request from the corresponding author. The data are not publicly available due to privacy restrictions.
